# Relationships between consumption patterns, health beliefs, and subjective wellbeing in Chinese Baijiu consumers

**DOI:** 10.1186/s13011-021-00369-8

**Published:** 2021-04-07

**Authors:** Debra Ann Metcalf, Anthony Saliba, Kirsty McKenzie, Appy Gao

**Affiliations:** 1 School of Psychology, Charles Sturt University, Locked Bag 588, Barooma Street, Wagga Wagga, NSW Australia; 2 Functional Grains Centre, Wagga Wagga, NSW Australia; 3 Graham Centre for Agricultural Innovation, Wagga Wagga, NSW Australia

**Keywords:** Chinese wellbeing, Subjective wellbeing, Alcohol consumption and wellbeing, Culturally-informed alcohol reduction programs

## Abstract

**Background:**

Alcohol consumption in China has increased rapidly, and there have been calls for policies and programs to address the issue. Alcohol plays a complex and important role in Chinese culture, where it is considered a symbol of happiness and is associated with wellbeing. Alcohol reduction policies may fail unless they take these cultural and social meanings of alcohol, and its relationship to wellbeing into consideration. Baijiu is a clear fermented spirit that is widely consumed in China and has strong cultural associations with health, wellbeing and prosperity. There is a lack of research on how consumption patterns relate to cultural beliefs and subjective wellbeing.

**Methods:**

An online survey of *n* = 1992 Chinese adults was conducted to determine frequency and volume of baijiu consumed; beliefs about health benefits and traditional importance; and associations with subjective wellbeing.

**Results:**

Higher frequency and volume consumed were associated with higher subjective wellbeing, controlling for age and income. We also found small to medium significant associations between consumption frequency and volume and: belief that baijiu is healthy; and that tradition dictated they should drink baijiu.

**Conclusions:**

The traditional and health values placed on baijiu, and its association with wellbeing, may help inform policy developments and alcohol reduction campaigns.

## Background

Alcohol consumption has increased rapidly in China in recent years, and has brought with it an increase in health-related and social problems [1]. It is estimated that 8.4% of men and .2% of women have an alcohol use disorder in China [2]; and there are very high rates of binge drinking, especially among men [2, 3]. China has less stringent policies around consumption of alcohol compared to other countries in the region [3] and there are few national alcohol laws or significant programs aimed at regulating alcohol consumption to reduce health and social problems [1, 2]. There is a clear need to reduce rates of alcohol consumption in China, and there have been calls for the government to address the issue through policy changes and public health campaigns [1, 3–5]. Newman [5] has argued that adopting a western-style approach to reduction in alcohol-related harms is likely to backfire if such policies interfere with traditional drinking practices, and suggests that a careful consideration of the role of alcohol in Chinese culture is essential in policy development. More broadly, the World Health Organisation [6] has argued that the development and implementation of policies to reduce alcohol consumption should be tailored to accommodate local contexts.

Jiu (alcohol) is inextricably linked to Chinese culture, and many Chinese believe that moderate consumption of alcohol is healthy and improves social relationships [7]. Alcohol is seen as a representation of happiness [5]; has cultural associations with career advancement and prosperity [4], and is an important element of business negotiations [8]. Alcohol consumption often occurs as part of complex toasting rituals at family, business and community meal gatherings and Chang [9] and others [8] have argued that consumption of alcohol in this context is central to the development and maintenance of relationships. According to Hwang [10], in Chinese culture effective relationships may help people maintain “psychological homeostasis” (p.171), and navigate systems of government and gain access to resources. Drinking may therefore perform important social and business functions for many Chinese, which may impact attempts by others to limit consumption [5, 8, 9].

Specific types of alcoholic beverages may also have cultural associations and meanings that may impact on attempts to promote responsible drinking [11]. In China, one such type of alcohol is Baijiu, a crystal clear alcoholic fermented spirit that originated in China, and that is one of the oldest distilled liquors in the world [12]. Baijiu is widely consumed throughout China, where 67% of alcohol consumed is in the form of spirits, including Baijiu [2]. Baijiu is inextricably entwined into Chinese culture and traditions and is considered to be China’s national drink [13, 14]. Many Chinese believe that consumption of a small amount of baijiu every day is good for health [14]. Historically, baijiu was the drink of choice at ritualised drinking parties through which individuals sought to establish and strengthen social ties, and attain creative and spiritual development [15]. Today, it remains popular in social situations such as the abovementioned toasting rituals at business, community and family meal gatherings through which personal and business relationships may be fostered and maintained [8, 13].

Because of the high cultural value placed on baijiu as a traditional and healthy product, and because it is consumed in contexts which may be associated with material and social benefits for individuals, understanding baijiu consumption in China may therefore inform the development and implementation of policies to address alcohol-related problems. Despite the strong cultural associations between baijiu and wellbeing, the authors could not identify any previous research linking the two. Geiger and MacKerron [16] note the relative lack of research on subjective wellbeing and alcohol consumption in general, and argue for the importance of a more complex understanding of this relationship to inform policy development.

Contemporary theories of subjective wellbeing provide a useful context in which to consider the potential relationship between subjective wellbeing and baijiu consumption. The theory of subjective wellbeing homeostasis suggests that subjective wellbeing is under genetically determined homeostatic control through processes that return it to a set point after changes in environmental conditions [17, 18]. Subjective wellbeing is theorised as being primarily an enduring, background mood state (happy, content, alert), and it is this mood state that is understood as being under homeostatic protection [18]. Several buffers (cognitive and external) operate to help maintain homeostatically protected mood near the set point when the environment becomes adverse. These include money, personal relationships, and a sense of purpose or meaning in life [18–20]. Factors such as lack of social support, poverty, and lack of meaning in life may lead to homeostatic failure when these are chronic and sufficiently severe [17, 20]. As discussed above, in China alcohol (including in particuar baijiu) is often consumed in contexts that facilaite the operation of buffers such as personal relationships and access to resources.

This article reports on research on the perceived benefits and cultural importance of baijiu consumption; consumption (frequency and volume); and whether consumption is associated with subjective wellbeing, measured using the Personal Wellbeing Index [21]. Subjective wellbeing has been found to be associated with income and with age in Chinese samples, with those on higher incomes and those in older age groups typically scoring higher on measures of subjective wellbeing [22, 23]. We therefore controlled for these variables when exploring the relationship between baijiu consumption and subjective wellbeing.

## Methods

### Procedure

The research was funded by the Grains Research and Development Corporation, and approval was given by the Charles Sturt University Human Research Ethics Committee. An online survey was administered via a market research company to gather data about Chinese adults’ attitudes and behaviours relating to baijiu consumption; and their subjective wellbeing. Prior to the development of the survey two of the authors conducted an extended trip to China involving consultation with academics and cultural experts, to gain an understanding of the cultural importance of baijiu, and to consider issues of cultural relevance and sensitivity.

The survey was administered in Chinese, the written form of the Chinese language being universal throughout the provinces and territories. The translation was performed by one of the authors, who is a Chinese National and a qualified translator. It was checked via a system of back translation. A pilot test was subsequently undertaken with a Chinese National who had no involvement in the research, who read the Chinese characters and explained their meaning to the authors. The questionnaire was then completed by the pilot study participant, after which the answers were discussed to ensure complete understanding of the nature and meaning of the questions. The translation of questionnaire items was deemed to be accurate and valid.

### Participants

Participants were all residents of China who expressed willingness to participate in online surveys and were paid a nominal sum in vouchers for their participation. Eligibility criteria required that participants were Chinese residents over 18 years of age, had consumed baijiu in the previous 12 months, and declared an active role in household purchasing decisions. Age range was 18 to 80 (*M* = 41.48 years, *SD* 13.24) with 961 male, 1029 females and 2 gender neutral participants completed valid questionnaires. The majority of participants were Chinese citizens (*n* = 1985, 99.65%), with the remaining participants either permanent residents, temporary residents, or work visa holders. Chinese language (including a dialect) was nominated as the main language spoken for all except 4 participants, for whom English was the main language spoken, and all provinces of China were represented with the exception of Tibet and Qinghai. An undergraduate degree or higher was held by 77.5% of the participants and 78% reported being in full time employment. Less than 1% reported being unemployed and the rest were either employed less than full time or retired. More than half of participants earned greater than the average annual income of 62,029 yuan pa [24] and the greatest represented household size was *3 or 4 people* (*n* = 1340, 67.3%).

### Apparatus

#### Personal wellbeing index – adult [21]

The online survey included the Personal Wellbeing Index – Adult (PWI); demographic items; items regarding Baijiu consumption (described below); as well as other measures not discussed here that were part of a larger survey on Baijiu consumption habits in China. The PWI is a commonly used domain-based scale that has been found to be a valid and reliable (Cronbach alpha between .70 and .85) measure of subjective wellbeing in a number of countries including China [21, 25]. The PWI measures seven domains that reflect the first-order deconstruction of the construct of overall satisfaction [17], measured on an 11-point rating scale with higher scores reflecting higher level of satisfaction. These domains include satisfaction with: standard of living, personal health, achieving in life, personal relationships, personal safety, community-connectedness, and future security [21]. An optional 8th item measuring satisfaction with spiritualty and religion was not included in the present study, as there is little evidence about the validity of this item in China, and most research has opted to omit it [26]. In the current study, the Cronbach alpha coefficient for the seven items was .91. An additional item on the scale measures satisfaction with life as a whole and is used as a regression construct to determine unique variance of PWI items.

Means for the PWI in Western countries fall between 70 and 80 [21], while means for China are typically around 10 points lower [25–27]. In the Chinese context, cultural factors such as modesty, eschewal of extreme emotional expression, and not wanting to tempt fate may help account for the lower means [19, 28, 29].

#### Consumption of baijiu (frequency and volume)

Participants were asked to indicate on a 7-point scale how often they consumed baijiu. Choices were; less than once every 3 months, once every 1–3 months, once per month, several times each month, once per week, several times each week, and every day. Participants were also asked to indicate the number of 750 mL bottles (or the equivalent) of baijiu they consumed in a typical month.

#### Perceived health benefits and cultural importance of baijiu

Participants were asked to rate the extent to which they agreed with the statement that tradition dictated that they should drink baijiu; and that drinking baijiu was healthy. Answers were on a 7-point rating scale, with 1 = strongly disagree and 7 = strongly agree. Participants were also asked to indicate the degree to which they considered Chinese culture an important reason for choosing to drink baijiu. Answers were scored on a 7-point scale from 1 = totally unimportant to 7 = extremely important.

### Data analysis

Collected data was downloaded from the market research company platform in a de-identified state, and analyses were carried out using IBM SPSS® version 24. Raw scores for the PWI were converted to the standard 0–100 scale format and 12 datasets were removed prior to analysis for consistently recording maximum scores, as recommended by the PWI manual [21]. Data were analysed using descriptive and inferential statistical procedures, including Pearson’s correlation (r) and Spearman’s rho (r_S_).

## Results

All variables were checked for statistical assumptions. Where assumptions were not met, appropriate non-parametric procedures were chosen.

Results indicated that baijiu was frequently consumed (once a week or more) by more than half of the participants (54.6%). Frequency of consumption is reported in Table 1.

**Table 1:** Frequency of consumption of Baijiu

Frequency	% (n)
Every day	4.8 (96)
Several times a week	30.8 (614)
Once a week	19.0 (378)
Several times a month	23.0 (459)
Once a month	7.2 (143)
Once every 1–3 months	8.4 (167)
Less than once every three months	6.8 (135)

*N* =  1992 Chinese adults were surveyed online regarding their consumption of baijiu. Participants were asked to indicate on a 7-point scale how often they consumed baijiu. Choices were; less than once every 3 months, once every 1–3 months, once per month, several times each month, once per week, several times each week, and every day

Volume of baijiu consumed was determined by asking participants how many 750 ml or equivalent bottles of Baijiu they personally consumed per month. Volume was also translated into the equivalent in standard drinks (based on 10 g alcohol) using the following formula: bottle size (.750) x alcohol content x gravity of ethyl alcohol (.789). Equivalent average daily standard drink consumption was based on a 31-day month. Because Baijiu varies in alcohol content, two calculations were made, one for 35% alcohol, and one for 60% alcohol. These findings suggest that 20.8% of Chinese are drinking more than 2 standard drinks a day, based on an alcohol percentage of 35%; and 33.7% are drinking more than 2 standard drinks a day based on an alcohol percentage of 60% (see Table 2).

**Table 2:** Number of 750 ml bottles (or equivalent) of Baijiu personally consumed per month

Number of bottles	Percentage (n)	Equivalent in standard drinks per month (day) (35% alcohol)	Equivalent in standard drinks per month (day) (60% alcohol)
Less than 1	28.4 (565)	Less than 20.7 (less than .6)	Less than 35.5 (less than 1.1)
1	17.4 (346)	20.7 (.6)	35.5 (1.1)
2	20.1 (401)	41.4 (1.3)	71 (2.2)
3	12.9 (257)	62.1 (2.0)	106.5 (3.4)
4	6.7 (133)	82.8 (2.6)	142 (4.5)
5	4.2 (83)	103.5 (3.4)	177.5 (5.7)
6	3.0 (59)	124.2 (4.0)	213 (6.8)
More than 6	6.9 (137)	144.9 or more (4.67) or more	248.5 or more (8 or more)

*N* = 1992 Chinese adults were surveyed online regarding their consumption of baijiu. Participants were asked to indicate the total volume of baijiu they drank in an average month, and were asked to indicate the volume as equivalent numbers of 750 ml bottles. Volume was translated into the equivalent in standard drinks (based on 10 g alcohol) using the following formula: bottle size (.750) x alcohol content x gravity of ethyl alcohol (.789). Equivalent average daily standard drink consumption was based on a 31-day month. Because Baijiu varies in alcohol content, two calculations were made, one for 35% alcohol, and one for 60% alcohol

There was a small to medium correlation between consumption frequency and income, *r* = .311, *p* < .001, and between consumption volume and income, *r*_*S*_ = .278, *p* < .001. Higher levels of income were associated with greater consumption frequency, and consumption volume, in the sample population. There was also a small correlation between consumption and age, with older age being associated with both higher consumption frequency (*r* = .184, *p* < .001) and higher consumption volume (*r*_*S*_ = .120, *p* < 001).

### Perceived health benefits and cultural importance of baijiu

We found high levels of agreement with statements about the cultural importance of baijiu, with 68.9% of participants agreeing with the statement that tradition dictates that they drink baijiu; and 79.4% agreeing that Chinese culture was important when choosing to drink baijiu. Fifty-two percent of participants agreed with the statement that drinking baijiu was healthy. Perceived cultural importance and health benefits variables were all positively associated with consumption frequency and amount (see Table 3), suggesting that higher levels of perceived cultural importance and belief in health benefits are associated with higher consumption. Of note, there was a medium effect size (*r* = .338, *p* < .001) for the association between the belief that drinking baijiu is healthy and frequency of consumption, indicating that 11% of the variability in frequency of consumption is associated with this belief. There was only a negligible and non-significant association between age and importance of culture when choosing to drink Baijiu, and associations with other health and cultural importance variables, although significant, were very small (see Table 3). Income had small to moderate positive associations with health and cultural importance variables, indicating that higher income is associated with higher belief that baijiu is healthy, and perceived cultural importance. All correlations were significant at *p* < .001 with the exception of age and tradition dictates that I should drink baijiu (*p* = .033), and age and cultural importance of baijiu, which was not significant (*p* = .271).

**Table 3:** Associations with health beliefs and cultural importance of baijiu

Belief	Frequency	Volume	Income	Age
Drinking baijiu is healthy	*r* = .338**	*r*_*S*_ *= .*298**	*r* = .243**	*r* = .081**
Cultural importance of baijiu	r = .111**	r_S_ = .119**	*rho* = .071**	*r*_*S*_ = −.025
Tradition dictates that I should drink baijiu	*r* = .266**	r_S_ = .249**	*r* = .154**	*r* = .048*

*r* = Pearson’s correlation*; r*_*S =*_ Spearman’s Rho; **p < .001; *p < .05

*N* = 1992 Chinese adults were surveyed online regarding their consumption of baijiu and their beliefs about its health benefits and cultural value. Participants were asked to rate the extent to which they agreed with the statement that tradition dictated that they should drink baijiu; and that drinking baijiu was healthy. Answers were on a 7-point rating scale, with 1 = strongly disagree and 7 = strongly agree. Participants were also asked to indicate the degree to which they considered Chinese culture an important reason for choosing to drink baijiu. Answers were scored on a 7-point scale from 1 = totally unimportant to 7 = extremely important. Correlations between beliefs about baijiu and: age, income, frequency of consumption and volume consumed per month were calculated using Pearson’s correlation, or Spearman’s Rho as appropriate. Positive associations indicate that higher belief is associated with higher age, income, frequency of consumption, and volume consumed

### Baijiu consumption and subjective wellbeing

Total mean converted (0–100) score recorded for the PWI in the current study was 73.53 (*SD* = 14.70). The mean was higher than reported in previous studies in China [26]. Individual domain scores are reported in Table 4.

**Table 4:** Means and Standard Deviations for the individual PWI domain items

Variable	***M***	***SD***
1. Standard of living	73.50	18.02
2. Health	73.98	18.05
3. Achieve in life	69.78	19.22
4. Personal relationships	73.32	18.16
5. Safety	75.60	17.95
6. Community	73.98	17.11
7. Future security	74.54	18.37

*PWI* Personal Wellbeing Index

The Personal Wellbeing Index The P (PWI) measures subjective wellbeing across 7 domains, on an 11-point rating scale with higher scores reflecting higher level of satisfaction in each domain.

Construct validity of the scale was assessed by regression of the seven items against the *satisfaction with life as a whole* item as described in the PWI manual [21]. All seven domains were found to make a unique contribution to the life as a whole domain; and inter item correlations were strong for all domains, as theoretically determined appropriate [21]. Correlation and regression statistics are reported in Table 5.

**Table 5:** Correlation coefficients and regression statistics for the Personal Wellbeing Index

Variable	LAW	1	2	3	4	5	6	B	β	Sr^**2**^
1. Standard of living	.77							.41**	.44	.084
2. Health	.66	.60						.19**	.20	.021
3. Achieve in life	.66	.66	.61					.09**	.11	.005
4. Personal relationships	.61	.61	.56	.64				.07**	.07	.002
5. Safety	.60	.60	.57	.57	.62			.04*	.05	.001
6. Community	.58	.56	.53	.59	.60	.62		.06*	.06	.002
7. Future security	.62	.62	.59	.62	.60	.68	.66	.04*	.05	.001

*p < .05, **p < .001

*LAW:* Satisfaction with *life as a whole*

The Personal Wellbeing Index measures subjective wellbeing across 7 domains. An eighth item measures satisfaction with life as whole. This item is included as a check of validity. Individual domains are regressed against this item to determine if they make a unique contribution.

Small positive correlations were found between total wellbeing (as measured by the PWI) and frequency of baijiu consumption (*r* = .266, *p* < .001); and volume of baijiu consumed per month (*r*_*S*_ = .279, *p* < .001).

As subjective wellbeing has been found to be associated with income and with age, partial correlation was used to explore the relationship between both frequency and volume of baijiu consumption and total wellbeing, while controlling for these variables. There was a small, positive correlation between baijiu consumption frequency and total wellbeing, after controlling for income (*r* = .205, *n* = 1986, *p* < .001), and age (*r* = .244, *p* < .001). Higher frequency of baijiu consumption was associated with higher reported subjective wellbeing. Because assumptions were not met for the volume variable, non-parametric procedures were used as described by Bradburn [30]. A weak, positive correlation was found between volume and total wellbeing after controlling for income (*r*_*S*_ = .217, *n* = 1975, *p* < .001), and age (*r*_*S*_ = .257, *p* < .001), indicating that higher volumes of baijiu consumption were significantly associated with higher subjective wellbeing after controlling for these variables.

## Discussion

This study investigated baijiu consumption in China, within the context of a need to understand the cultural and social importance of alcohol in China, and how this relates to subjective wellbeing. Over half the participants in the present study reported drinking baijiu once a week or more, and large numbers consumed more than two standard drinks a day (based on 10 g alcohol (for a discussion of differences in alcohol content in standard drinks internationally, see Kalinowski and Humphries [31]. Given consumption levels reported here and elsewhere [1–3], there is clearly a need for policy and interventions that ameliorate alcohol-related harms in China. However, our research also indicates that alcohol-reduction campaigns run the risk of being incongruent with views held by many Chinese. We found that just over 50% of Chinese think that drinking baijiu is healthy. Research conducted in other contexts [32] has found that the belief that wine is healthy is associated with an increase in frequency of consumption, but not volume. The present research found a medium association between belief that baijiu is healthy and both frequency and volume of consumption. This indicates that this belief may play a role in overconsumption of baijiu in China, and highlights the importance of culturally specific research into this issue. We also found small associations between perceived cultural importance of baijiu, and the belief that culture dictated that one should drink baijiu, and consumption.

In addition, we found small associations between baijiu consumption (frequency and volume) and wellbeing, after controlling for income and age. This should not be taken to suggest that baijiu consumption is directly associated with or causes positive wellbeing. Instead, it is more likely that baijiu is being consumed in contexts that foster wellbeing (for example through promoting positive relationships and access to material resources) in the Chinese context. This suggestion is drawn from Chang [9] and Szto [8], who have argued that toasting rituals perform important social and business functions for many Chinese. Chang [9] also noted that it may be virtually impossible for some Chinese to drink less, even though they may want to, because of these important social functions; and campaigns aimed at reducing alcohol consumption should find ways for such social functions to be maintained [8, 9]. Overall, our results therefore support previous contentions [5, 8, 9] that efforts to curb consumption may be counter-productive unless they take the long-standing values and practices associated with baijiu consumption into consideration. Szto [8], for example, discusses the dangers of ignoring drinking rituals in business negotiations, and argues for the need for inter-disciplinary forums on alcohol reduction, and culturally specific interventions that recognise and value traditional customs while promoting moderation.

It is worth noting that the total mean converted (0–100) score for the PWI in the current study was 73.53 (*SD* = 14.70, *n* = 1992). This is higher than previously reported for Chinese-speaking populations, where means typically fall between 60 and 70 [25–27]. This may reflect the sampling strategy, which involved third-party recruitment via a database of individuals who had indicated an interest in participating in online research. Potentially, such individuals may have been less impacted by cultural response biases that have been suggested to account for the lower means in China [19, 28, 29]. It is also worth noting that in the current study, our sample was skewed towards those with higher than average incomes; in full employment; and with a university degree or equivalent. China has experienced rapid and significant economic and social transformation in the past decades, and wellbeing is becoming more associated with individualistic values [33]. Further research could help to determine if the present high mean for the PWI is an anomaly, or if it instead reflects wider changes in Chinese society. Such changes have resulted in improvements in material conditions for many, and may potentially be associated with decreased salience of cultural response biases associated with collectivist culture (e.g. modesty) that lead to lower means on the PWI. This may be most apparent in the population of middle class affluent Chinese (such as that represented in the present sample) who have benefited from economic changes and are at the forefront of social change and most exposed to Western ideas.

### Limitations and recommendations

The current study found small associations between subjective wellbeing and baijiu consumption in China, controlling for income and age. Ascertaining why these associations occur will require a more complex design that takes into consideration the possibility of reciprocal relationships, and that includes variables not accounted for in the present study. One such variable may be opportunities for social connection, which is intimately linked to alcohol consumption in Chinese culture and which may contribute to subjective wellbeing by fostering affective and instrumental relationships. Further research is needed to explore this.

The sampling strategy may also have impacted on results. Participants were recruited through an online panel and were requited to have consumed baijiu within the last 12 months. It is possible that participants may have had more positive views about baijiu than would be found in the general population.

## Conclusion

Alcohol consumption, and in particular the consumption of baijiu, has cultural associations with health, happiness and prosperity in Chinese society. Alongside this, alcohol consumption is leading to significant social and health-related problems. It has been argued that attempts to address the growing social and health problems associated with alcohol consumption in China must take local customs and values into account. The present research aimed to understand baijiu consumption in China in this regard. Traditional and health values accorded to baijiu were associated with higher consumption. Furthermore, higher consumption was also modestly associated with higher subjective wellbeing. It is beyond the scope of the present research to fully account for these associations. It is unlikely, however, that baijiu makes a direct contribution to wellbeing. Instead, we suggest that this result may reflect the fact that baijiu is consumed in contexts which contribute to material and social wellbeing for individuals. A greater understanding of the relationships found in the present research may benefit the development of harm reduction campaigns. To gain traction, such campaigns may consider working alongside traditional values and practices, not against them.

## Data Availability

The datasets generated and/or analysed during the current study are not publicly available because the intellectual property is owned by the funding body. They may be available from the corresponding author on reasonable request subject to approval from the funding body.

## Abbreviation

PWI: Personal Wellbeing Index
